# Forces generated in stabbing attacks: an evaluation of the utility of the mild, moderate and severe scale

**DOI:** 10.1007/s00414-017-1702-7

**Published:** 2017-10-16

**Authors:** Gary Nolan, Sarah V. Hainsworth, Guy N. Rutty

**Affiliations:** 1 East Midlands Forensic Pathology Unit, University of Leicester, Robert Kilpatrick Building, Leicester Royal Infirmary, Leicester, LE2 7LX UK; 2 Department of Engineering, University of Leicester, Leicester, UK; 3 Present Address: School of Engineering and Applied Science, Aston University, Birmingham, B4 7ES UK

**Keywords:** Stab wound, Force, Instrumented knife, Dynamometer, Knife, Screwdriver, Blunt force

## Abstract

The commonest way of killing in the UK is by a sharp instrument. Knight reported in 1975 that it is impossible to discern with any degree of certainty the degree of force used to create a stab wound. Despite this, expert witnesses continue to approximate the degree of force used for their reports and evidence in court. It is usually subjectively categorized as mild, moderate or severe, based solely on the examination of the wound. We undertook a study considering forces generated in a range of blunt trauma actions, using a novel force plate dynamometer to measure the peak forces obtained by adult male and female volunteers. We then studied forces generated by stabbing skin simulants and porcine samples with knives and screwdrivers. Men generated more force than women during stabbings which was found to be equivalent to somewhere between the blunt trauma actions of pushing a button to a single-handed push. When asked to stab using what they thought was mild, moderate and severe force, although volunteers were able to actively decide the force used, the actual force was found to be influenced by the weapon, sex of the individual, hand used and biological/anatomical site penetrated. This study shows that the forces generated by volunteers in mild, moderate and severe stabbing tests in almost all cases were significantly greater than the forces required for skin penetration. We suggest that the use of subjective force scales is inappropriate. Rather than use of a subjective scale, we suggest that the force required in any stabbing requires investigation in four areas: the tip radius of the weapon, minimal force required for penetration, the sex of the assailant and whether the force required for penetration is greater than that that can be generated by a person stabbing. This allows for the use of an evidence-based two-tier scale to suggest the force required.

**Electronic supplementary material:**

The online version of this article (10.1007/s00414-017-1702-7) contains supplementary material, which is available to authorized users.

## Introduction

Knife crime continues to be a concern worldwide, particularly in those countries where access to guns is restricted. Between October 2014 and September 2015, the police of England and Wales recorded 188 homicides involving a knife or sharp instrument (33% of all homicides). A sharp instrument is defined as any object that pierces the skin [1]. Weapons encountered in such attacks include kitchen knives, utility knives, sheath knives, penknives, scissors, samurai swords, bayonets, screwdrivers and glass bottles. Expert witnesses are often requested to approximate the degree of force used to cause the stab wound, as part of their statement and evidence provided to a court. The common practice is to estimate a penetrative force using a subjective scale of mild, moderate or severe. Some use a fourth point on the scale of “extreme”. This is often based solely on the examination of the resultant wound [2, 3]. A subjective visual assessment of the sharpness of the blade may also be taken into account. This evidence has the potential to influence the court in its consideration of the stabbing incident.

Studies have been conducted previously to investigate the force involved in stabbings. The earliest of these was by Knight in 1975, who found it impossible to answer the question with any degree of certainty [4]. Research conducted in this area to date has focused on the use of destructive techniques, for example the use of scanning electron microscopy (SEM) or measuring force using methods that are not suited to use with forensic evidence because they rely on changing the evidence from that which was used in the incident. The use of “instrumented” knives has been favoured by researchers, which typically have a load cell inserted between the blade and the handle to measure forces via a long cable from the handle to a computer [3–13]. However, the increased weight of the knife and the cable can interfere with the participants’ ability to perform a “natural” stab. Engineering assessment of the knife’s sharpness can give a better quantitative measurement of the knife’s penetration ability, but this needs to be understood in the context of the forces that can be generated by people in stabbing attacks [2].

In this study, we used a novel dynamometer to investigate the forces generated by adult males and females using a variety of sharp force weapons to cause stab wounds and compared these to a range of force generating hand actions and the forces required for the weapons to penetrate test material. The aim was to consider whether the use of the commonly used subjective scale is appropriate when providing evidence to a court concerning the possible forces used in stabbings, or whether a more scientific approach should be adopted by investigating the penetrative potential of the weapon itself.

## Methods

### Study design and ethics

This is a single-centre, prospective study undertaken as part of a PhD research program [14]. The study was approved by the University Research Ethics Committee (University of Leicester ref.: gn41-18e4). All participants were volunteers recruited from the University by advertisement.

### Procedures

#### Instrumented knife

As instrumented knives are often used in such experimental research, one was manufactured using an all-purpose (V Sabatier) knife in the Department of Engineering at the University of Leicester as described in on-line appendix, p2. This was used as a control for the use of the novel dynamometer.

#### Dynamometer

**Fig. 1:**
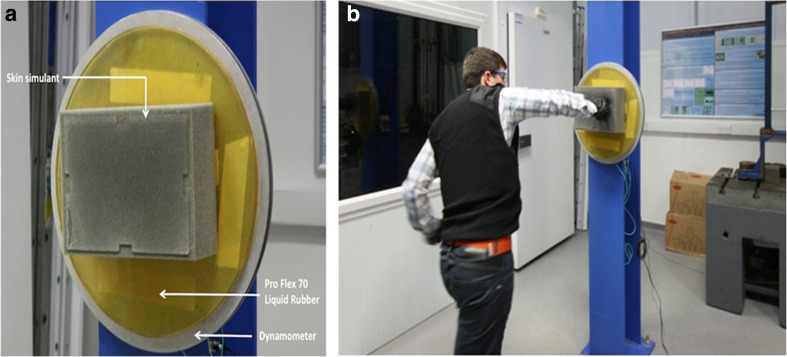
**a** The dynamometer with a skin simulant attached. **b** An example of a volunteer stabbing a knife into the dynamometer

A dynamometer is a device consisting of an instrumented plate that is used for dynamic measurement of force. This approach has the advantage that the force generated by a range of actions such as pushing, punching or slapping can be measured along with stabbing actions from knives and other penetrating implements such as screwdrivers. A purpose-built dynamometer was developed for the study as detailed in on-line appendix, p2 (Fig. 1a).

#### Calibration

Before testing, the dynamometer was calibrated by standing weights of known mass on the dynamometer and adjusting the load cells to give the correct reading.

#### Safety

Figure 1b shows a volunteer stabbing the dynamometer. In order to ensure that the participants of the stab testing were safe, the participants were required to wear personal protective equipment as described in on-line appendix, p2. The safety procedures were subject to a full risk assessment.

#### Skin simulants

Due to difficulties in undertaking research using human biological material, no human skin or body parts were used for the study. Rather, human skin was substituted with a skin simulant; the manufacture of which is detailed in on-line appendix, p2. Pork leg and pork ribs were also used to provide two different biological, anatomical area models of the human body.

#### Weapon penetration

Prior to the volunteer tests, a Materials Testing System (MTS, Hounsfield Test equipment Ltd., Surrey, England) was used to provide the minimal penetration forces required for a sample of five all-purpose (V Sabatier) knives (Richardson Sheffield, Sheffield, UK), five steak knives (Chef Aid, Suffolk, UK), the instrumented knife (modified all-purpose (V Sabatier) knife (Richardson Sheffield, Sheffield, UK)), a flat-headed screwdriver and a Phillips-headed screwdriver (both manufactured by Stanley, Berkshire, UK) to penetrate pork leg and pork ribs. Each weapon was tested three times. The blunt edge and tip radius as well as the blunt tip angle were calculated for each weapon.

#### Blunt trauma

A cohort of five male and five female volunteers were asked to undertake a series of actions to apply blunt trauma to the dynamometer. Each action was explained to the volunteer but was open to their interpretation. First, they pushed the dynamometer using one finger (button push) as they would a button in a lift, a door bell or a light switch, etc. Then, they pushed using one arm as if pushing or opening a door. This was in contrast to the next push with both arms, which was to be carried out as if the participants were trying to push someone over. Next, they are asked to do a slap. They stood facing the side of the dynamometer and slapped it as though slapping someone across the face, for example a slap you “may” give someone who had offended you. The reason for not positioning the slap as a slap as hard as you can was due to initial testing finding that individuals were unaware of how hard they could slap but could relate to slapping someone across the face. Finally, they punched the dynamometer as hard as they could. They were requested to perform each of the actions three times using each hand (except the push with both arms).

#### Stabbing

Two different sets of volunteer experiments were undertaken. First, the cohort of volunteers who had undertaken the blunt trauma experiments were asked to stab the skin simulant as hard as they could with each hand using an all-purpose knife, steak knife, flat-headed screwdriver and a Phillips screwdriver (stabbing experiment 1). The volunteers were then invited to repeat the stabbings, this time using pork leg and then pork ribs instead of skin simulant. Each stabbing experiment was repeated three times per hand per weapon per volunteer per simulant.

A second cohort of volunteers, five males and five females, blind to the actions and results of group 1, were asked to stab pork leg and pork ribs. Again, volunteers were asked to carry out three stabs with an all-purpose knife, steak knife, flat-headed screwdriver and a Phillips screwdriver but this time they were asked to try and stab with what they felt was a mild, moderate or severe force (stabbing experiment 2). The judgement of force was completely done at their own discretion. They were given no guidelines.

#### Data collection

Test data was obtained using a LabView program (National Instruments, Newbury, England) that allowed recording of the force versus time. The participant was asked to prepare to stab; they were given a countdown of “3, 2, 1” at which point a remote trigger was pressed. The LabView program then recorded 3 s of data. Each 3-s period collected 30,000 data points. The LabView program plots an amplitude (where amplitude is force) versus time plot. A typical output is shown in the on-line appendix, p4. The data was recorded as a .csv file. Data analysis was performed using Microsoft Excel.

### Outcomes

The primary end point of this study was to provide quantitative data for adult males and females through the use of the dynamometer for forces generated through a range of blunt trauma hand actions and stabbing actions. The dynamometer data was compared against that of a known testing method, i.e. that of an instrumented knife and the minimal penetrative force of each weapon assessed using the MTS. This data was then used to consider whether the use of the subjective scale commonly used by court witnesses is appropriate to provide evidence to a court.

### Statistical analysis

Statistical support was provided by the University of Leicester Bioinformatics and Biostatistics Analysis Support Hub (B/BASH). The data was analysed using descriptive and mixed effects linear regression statistics.

### Role of the funding source

The study was funded by internal resources. The investigators had full access to all the data in the study and were responsible for the decision to submit the manuscript.

## Results

The volunteers were a mixture of male and female with ages ranging between 21 and 52 and with heights and weights representative of a random sample from the Leicester University population. The body data of the two volunteer cohorts is presented in on-line appendix, page 5. The individuals undertook a total of 3204 stabbing experiments which generated 96,120,000 data points for analysis.

### Weapon penetration force

The individual penetrative force for skin simulant, pork thigh and pork ribs as well as the blunt edge radii, tip radii and blunt edge tip angle for five steak knives and five all-purpose knives of the type used in the volunteer experiments as well as the flat-headed and Phillips-headed screwdrivers and the instrumented knife were investigated. The data is presented in the on-line appendix, p7. The average for the individual weapon penetrative forces for each simulant was calculated to provide a baseline against which the volunteer generated forces were considered (Table 1). The individual tip, edge and force measurements were found to vary within each weapon type and the average individual penetrative forces were found to be weapon and material/biological location dependent.

**Table 1:** Average minimal force (A Min F), average maximum force (A Max F) and average force (AF) for all individual penetrative forces for each weapon type for each simulant type in Newtons (N). The individual minimal, maximum and average forces as well as the blunt edge radius, tip radius and blunt edge tip angle for each weapon are found in Tables 3, 4 and 5 in the on-line additional resource data

	Skin simulant			Pork leg			Pork rib		
Weapon	A Min F	A Max F	AF	A Min F	A Max F	AF	A Min F	A Max F	AF
Sabatier knife	11.4	13.8	12.6	10.7	13.8	12.1	10.1	13.5	12.0
Steak knife	18.7	20.9	19.8	40.2	47.8	41.2	38.0	47.3	41.9
Instrumented knife	12.7	13.9	13.4	25.4	25.5	25.4	28.7	32	30.9
Flat-headed screwdriver	63.2	78.6	73.3	Not achieved	Not achieved	Not achieved	Not achieved	Not achieved	Not achieved
Phillips-headed screwdriver	60.0	67.6	63.4	219.5	247.3	234.3	Not achieved	Not achieved	Not achieved

#### Blunt trauma

**Fig. 2:**
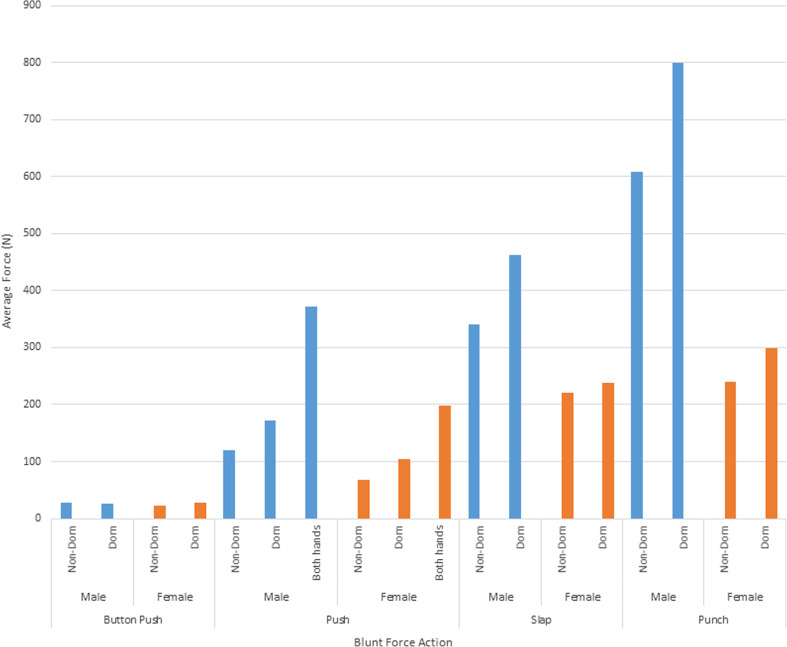
Mean force generated by blunt trauma actions performed by volunteers with their dominant and non-dominant hands

Table 2 and Fig. 2 show the results of the blunt trauma experiments. The results show, as would be expected intuitively, that, except for the male participant push button force, males generate more force than females and the forces generated for males and females are greater with their dominant hand. The greatest force generated was during a punch action followed by a slap, a push with two hands, a single-handed push and finally the least with a push button action. These observations provide comparative blunt trauma actions to consider the stabbing experiments against. For the results generated from the MTS experiments, the average force required for the all-purpose knife to penetrate all three simulants (Table 1) was less than that required to push a button with a finger and for the steak knife lay somewhere between pushing a button to a single-handed push.

**Table 2:** Forces generated in Newtons by blunt trauma actions by sex by hand

			Force			
Action	Sex	Hand	Mean	Std. dev.	Min	Max
Button push	Male	Non-dom	27.3	3.2	22.6	31.2
		Dom	27.0	5.6	19.3	34.6
	Female	Non-dom	23.5	4.9	15.2	27.0
		Dom	27.3	6.9	17.1	35.1
Push	Male	Non-dom	120.3	21.7	90.6	142.2
		Dom	171.6	100.3	82.2	331.7
		Both hands	372.4	187.0	108.4	599.7
	Female	Non-dom	67.7	12.4	51.3	83.1
		Dom	103.8	74.9	50.3	234.6
		Both hands	199.1	123.3	65.6	379.6
Slap	Male	Non-dom	339.9	87.3	256.7	468.8
		Dom	461.5	161.3	255.1	605.8
	Female	Non-dom	220.1	111.0	78.5	367.7
		Dom	239.0	95.1	103.2	355.9
Punch	Male	Non-dom	607.4	105.6	483.6	751.4
		Dom	800.0	85.8	656.9	871.7
	Female	Non-dom	240.6	72.5	134.6	319.1
		Dom	298.3	105.4	157.7	419.8

#### Stabbing experiment 1

**Fig. 3:**
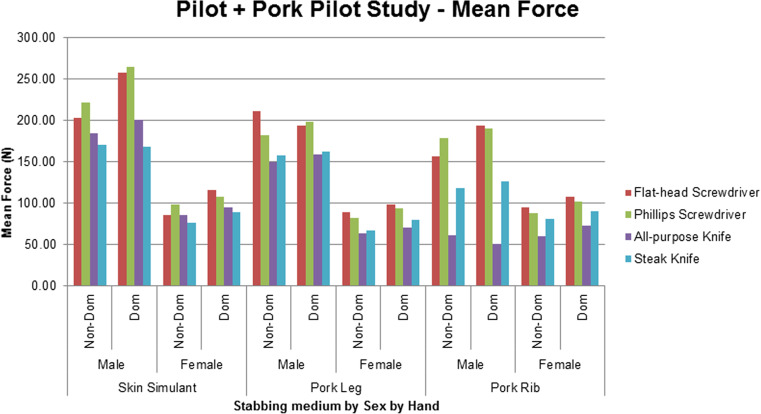
Mean force generated (Newtons) by males and females with each implement, by dominant and non-dominant hand with the skin simulant, pork thigh and pork ribs

Figure 3 shows the mean forces generated by the volunteers stabbing the skin simulant, pork leg and pork ribs as hard as they could be using an all-purpose and a steak knife as well as a flat-headed and Phillips-headed screwdriver. The minimal, maximum and standard deviation data is presented in the on-line appendix, p10. Male participants again generated more force than the females. The force generated with the dominant hand was greater than the non-dominant hand, with the only exception being for male participants using the steak knife where the force generated by the non-dominant hand was 2 N higher than that of the dominant hand. The mean forces generated in stabs using the screwdrivers were higher than those of the knives. When compared to the forces generated to the blunt trauma actions, the mean forces lay somewhere between those generated by a push with one hand and a slap.

Unfortunately, during testing with the pork leg, participant 2 broke both available all-purpose knives at the front bolster. This resulted in participant 9 being unable to carry out any tests using the all-purpose knife for pork leg or ribs, and participants 2, 3, 5 and 6 being unable to carry out any tests using the all-purpose knife for pork ribs. This accounts for the lower than expected mean forces. In the pork testing, as had been encountered with the MTS, some stabs with the screwdrivers did not penetrate the pork as follows; participant 3 for the Phillips screwdriver with the dominant hand for pork, participant 6 for the Phillips screwdriver with both hands for pork leg, participant 8 for flat-headed screwdriver for pork ribs and participant 10 for the flat-headed screwdriver and Phillips screwdriver for both hands for pork leg.

#### Stabbing test 2

**Fig. 4:**
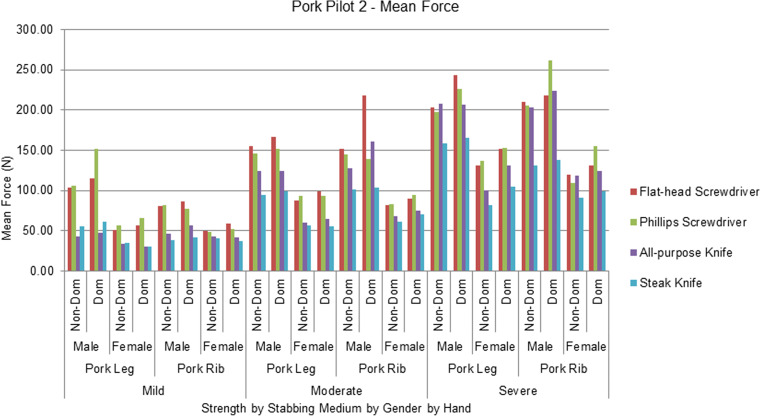
Mean force generated (Newtons) by males and females undertaking mild, moderate and severe force stabbings with each implement, by dominant and non-dominant hand with pork thigh and pork ribs

Figure 4 shows the mean forces generated by the volunteers stabbing the pork leg and pork ribs using an all-purpose and a steak knife as well as a flat-headed and Phillips-headed screwdriver whilst using their perceived idea of mild, moderate and severe force. The minimal, maximum and standard deviation data is presented in the on-line appendix, p12. It shows that the volunteers were able to generate stabs with varying levels of force, and that a stepped increase in the force from mild to moderate to severe in the stabbings for both types of stabbing medium (pork leg and pork ribs) was able to be produced for both hands and both sex. For mild force for both the steak and all-purpose knives with both hands, males stabbed at a force above the MTS minimal average penetrative force. Females on the other hand, with both knives, were able to cause penetration with mild force below the minimal average force identified with the MTS. When compared to the forces generated to the blunt trauma actions, the mean mild forces lay somewhere between those generated by a push button with one finger and push with one hand.

#### Statistical analysis

Representative examples of the results for the statistical analysis are found in the on-line appendix, p16. Both stabbing experiments identified significant differences in the forces generated between males and females as well as, for all but one of the analysis, dominant versus non-dominant hand experiments. Significant differences in the forces were seen between knives and screwdrivers. Strength and the biological material into which it was been stabbed was also found to be a significant factor. The only results that were not statistically independent were those with weapon 1. Weapon 1 was a comparison of the results obtained with a flat-headed and Phillips-headed screwdriver. The results show that there was overlap between the forces generated with each weapon and therefore that the force required to stab with these cannot be statistically separated.

## Discussion

The key issue in deciding whether a weapon creates a stab wound during a stabbing attack is whether the threshold force for penetration of the skin with that particular implement is met. Previous published work suggests that skin penetration typically requires ~ 35–55 N depending upon the weapon involved [12]. From the previous work of Hainsworth et al. [2], the ability for a weapon to penetrate skin is primarily dependent upon the tip radius of the weapon. Skin is known to provide the most resistance to penetration with muscle and fat being more readily penetrated. Thus, once the skin is penetrated, assuming that a force continues to be applied, then the weapon will continue to penetrate to a depth dependent upon the length of the weapon. This penetration can be altered by the weapon encountering cartilage or bone. For a knife to pass through cartilage, it has been reported that this may require 140 N and for the sternum 200 N [6]. All of these suggested values will be dependent upon the knife used and will differ from knife to knife even when manufactured by a single company [12]. Our study also identifies the importance of considering the sex of the assailant, their strength and the hand used to inflict the injury.

The data presented in our first stabbing experiments showed that for an unrestrained stab by a man, using their dominant hand, the mean peak force generated was approximately 150 N for pork thigh and considerable less for pork rib. This force is over three times that previously published for skin penetration and in the case of our baseline MTS data, for the all-purpose knife, over 12 times greater than actually required for pork penetration. For the same experiments, female volunteers generated lower forces than the males (approximately half the force generated by a male) but these forces were still greater than those required for skin penetration. Had we applied a mild, moderate or severe scale to these values then although the volunteers were asked to stab the dynamometer as hard as they could, i.e. severe force, the female volunteers, based on the results from experiment 2, would have stabbed at moderate force with the equivalent force for males would be somewhere between mild to moderate.

The volunteers were able to generate a scale of force, i.e. undertake what they considered to be mild, moderate and severe stabs. Thus, this study shows that it is possible for a person to actively decide the force which they wish to undertake a stab although there are multiple factors that influence the actual force used such as the weapon, the sex of the offender, the biological/anatomical site and the hand used.

Thus, based on the results of both experiments, in the case of a wound tract involving skin and organs only, applying a subjective scale of mild to moderate to severe force in an attempt to suggest to a court that one can differentiate degrees of force by simply looking at the resulting wound is inappropriate. All that can actually be said is that the minimal force required for that particular implement to breach the skin and cause an injury to skin and soft tissue only has been met.

If bone is penetrated, then, based on previous published data, it can be justified to suggest that greater force was used to inflict the injury. Considering the second set of stabbing experiments then this would be at the upper end of the volunteer’s perceived mild, moderate and severe force.

However, as this force is less than that of a double handed push, a slap of annoyance or a punch to suggest a stab wound through bone is “severe” force questions then how to describe a slap which may result in nothing more than a bruise but can be delivered at over twice the force. It is thus not sensible to use the same subjective mild, moderate and severe force scale to apply to both blunt and sharp force trauma, as currently occurs, despite the area of injury over which the force is applied being very different, i.e. comparing an impact with a fist to that of a tip of a knife. Sharkey et al. have reported that forces of 4000 N are required to cause a laceration to the scalp [15] by blunt force which far exceeds the force for penetration of the skin by a knife or the forces generated by stabbing during these experiments. Recently, Bolliger et al. have reported that the pocket knives used in their experiments required 906–1198 N to penetrate ribs, values which would not have been predicted based on previous published data and ones which demonstrate the importance of considering the properties of the weapon rather than the resulting wound [16].

Our results support the use of a sharp wound specific, two-tiered approach to commenting on force would be reasonable, i.e. above the minimal force required to breach skin and above the minimal force to penetrate bone. Our results support that a proper forensic investigation is required in all cases of stabbing to determine whether or not the tip of the weapon permits penetration and at what minimal force. If the tip of the weapon causes penetration at a force in excess of that expected for a stab wound in skin, then it is reasonable to suggest, as with penetration of bone, that greater force than expected was required with the implement to cause the injury. Such terminology, supported by scientific testing, may be more appropriate for court use than the current subjective scale.

These results have significant implications for the legal presentation of stab forces in court. Typically, a mild, moderate and severe subjective scale is used to suggest the level of violence during a stabbing attack based on the sole examination of the resulting wound. Some go further using a 4-point scale with “extreme” as the fourth subjective category. The data in this study does not support the use of such subjective scales especially as it could influence the court in its consideration of the case. We are of the opinion that the force required in any stabbing requires investigation in four areas: the tip radius of the weapon, minimal force required for penetration, the sex of the assailant and whether the force required for penetration is greater than that that can be generated by a person stabbing. This later requirement is unlikely to be made available to an enquiry but can be based upon published data such as illustrated within this paper. The dynamometer we have described in this paper can facilitate such considerations. By taking such an approach and applying an approach of “minimal force required reached” and if bone is involved, “a force greater than required to breach the bone”, this provides a more scientific approach to the presentation of expert testimony related to the force required to enact a stabbing.

## Electronic supplementary materials

ESM 1 (DOCX 390 kb)
