# Supplementary material for: Forces generated in stabbing attacks: an evaluation of the utility of the mild, moderate and severe scale
Source: Int J Legal Med. 2017 Oct 16;132(1):229–36. doi: 10.1007/s00414-017-1702-7 (PMC5748396; doi:10.1007/s00414-017-1702-7)
Supplement: Supplementary file 1 [file 414_2017_1702_MOESM1_ESM.docx]

# Supplementary Text and Tables to Nolan, Hainsworth and Rutty. Forces generated in stabbing attacks; a re-evaluation of the utility of the mild, moderate and severe scale.

## Contents

|  |  | Page |
| --- | --- | --- |
| Supple. Text | Expanded Methods | 2-3 |
| Supple. Figure 1 | An amplitude time plot obtained from the force plate dynamometer | 4 |
| Supple. Tables 1 and 2 | Volunteer body data | 5-6 |
| Supple. Tables 3,4 and 5 | Penetrative and weapon blade data | 7-9 |
| Supple. Table 6 | Force data for stabbing experiments 1 | 10-11 |
| Supple. Table 7 | Force data for stabbing experiments 2 | 12-15 |
| Supple. Text | Statistical analysis data | 16-19 |

## Supplementary text: Expanded methods

#### Instrumented Knife

The knife was cut between where the handle meets the blade, at the front bolster. The blade and the handle were threaded to allow for a load cell to be attached. The load cell used was a DLC101-1K dynamic load cell (Omega Engineering, Manchester, England). This was connected to an ACC-PS3-230VAC ac powered accelerometer amplifier and power supply using an ACC-CB4-15 15 foot BNCM/BNCM cable (Omega Engineering, Manchester, England). The amplifier was connected to a National Instruments Data Acquisition (DAQ) Card [NI USB-6221], (National Instruments, Newbury, England) using an ACC-CB2-10 10-32 BNCM cable (Omega Engineering, Manchester, England). The load cell used for the instrumented knife was chosen as it could measure dynamic forces of up to 1,000 N over a broad frequency range. The DAQ card was connected to a PC where all data collection and manipulation was managed through the LabView program that also recorded dynamometer data.

### Dynamometer

The dynamometer developed for the study consisted of a frame of two 6mm thick circular plates of aluminium (reduced weight of instrument) between which three piezoelectric load cells are placed (figure 1a). The piezoelectric load cells used (9313AA1) allow dynamic data capture (Kistler, Hampshire, England). They were chosen to accommodate a wide range of forces from 0 to a maximum of 5,000N, which can be produced from punching in boxing^17-19^. The piezoelectric transducer's measuring accuracy is practically constant over a wide range of forces. A single channel amplifier (ICAM 50731511) was used to allow the dynamometer to handle both large and small forces with equal sensitivity (Kistler, Hampshire, England). Movement of the aluminium plates was restricted to only one plane by the placement of the load cells, which were attached directly to the plates. A mount meant it could be moved, allowing testing of the so-called “walking into the knife” scenario that is sometimes presented in court as the causation of the stabbing.

### Health and Safety

In order to ensure the participants of the stab testing were safe, a disk made from ProFlex 70 liquid rubber was placed on top of the dynamometer to stop volunteers hitting the aluminium faceplate of the dynamometer, as this could result in bending or breaking of the knife, causing injury. ProFlex 70 was selected as during testing it was shown to allow some penetration of the knife but decelerated it to a stop fairly sharply. All volunteers were required to wear personal protective equipment that allowed free movement, including a stab vest, safety glasses or a full-face visor, arm guards and slash resistant gloves. The gloves had rubber palms that allowed for a good grip to be obtained on any knife handle. The safety procedures were subject to a full risk assessment.

### Skin Stimulants

This comprised of a silicone rubber layer bonded to open cell polyether foam. The foam was an open-cell polyether foam with a foam hardness in the region of 125–155N and a density of 23– 28kgm^−2^ (Acoustafoam, Shropshire, England). The silicone rubber used comprised of two parts mixed in a 1:10 ratio. 150g of the base (part B, transil 40) was mixed with 15g of catalyst/curing agent (part A, transil 55) for between 3-5 minutes (Mouldlife, Newmarket, England). Once the correct consistency was achieved the mixture was poured into a specially designed mould and spread out evenly to create a thin uniform layer. The polyether foam was then placed on top of the mould before the final set, to create a good bond between the skin and foam. The foam was then weighted with 6 x 1kg weights equally distributed over the foam block and left to set for 24h. This encouraged optimal bonding between the silicone and foam, while also minimising the formation of bubbles in the silicone. The thickness of the silicone layer was controlled by the mould and was repeatedly 1.5mm in thickness. The dimensions of the foam blocks were typically 500mm x 500mm x 100mm.

## Supplementary Figure 1: An amplitude time plot obtained from the force plate dynamometer. The dynamometer records for 3 seconds and the stab event is shown as the deviation from the zero amplitude line. The amplitude axis was calibrated with force, thus a peak amplitude of 124N correlates with a peak force in this stab of 124N. This stab was made with a bread knife.

## Supplementary Tables 1 and 2: The body data for the two volunteer cohorts.

Appendix Table 1. Blunt force and stabbing experiment 1 volunteer body data.

| **Participant Number** | **1** | **2** | **3** | **4** | **5** | **6** | **7** | **8** | **9** | **10** |
| --- | --- | --- | --- | --- | --- | --- | --- | --- | --- | --- |
| **Healthy Body Fat (%)** | 7.6 | 14.1 | 16.2 | 17.7 | 10.4 | 38.7 | 29.3 | 30.2 | 37.1 | 18.7 |
| **Weight (kg)** | 71.1 | 76.4 | 72.9 | 84.8 | 71.1 | 76 | 74 | 62.2 | 68.5 | 48.3 |
| **Healthy Body Water (%)** |  | 62 | 59.6 | 57.3 | 63.3 | 45.9 | 51.6 | 51.7 | 46.8 | 60.8 |
| **Visceral Fat** | 1 | 3 | 3 | 3 | 1 | 4 | 3 | 2 | 5 | 1 |
| **Muscle Mass** | 62.4 | 62.1 | 58 | 66.3 | 60.5 | 44.2 | 49.7 | 41.2 | 40.9 | 37.2 |
| **Physique Rating** | 8 | 5 | 5 | 5 | 5 | 2 | 5 | 5 | 2 | 8 |
| **Bone Mass (kg)** | 3.3 | 3.2 | 3 | 3.4 | 3.2 | 2.4 | 2.6 | 2.2 | 2.2 | 2 |
| **BMR (kcal)** | 1905 | 1917 | 1806 | 2063 | 1859 | 1471 | 1584 | 1346 | 1358 | 1208 |
| **Metabolic Age** | 12 | 16 | 19 | 23 | 12 | 39 | 31 | 29 | 41 | 12 |
| **Energy (kJ)** | 7970 | 8020 | 7556 | 8631 | 7778 | 6154 | 6627 | 5631 | 5681 | 5054 |
| **Age** | 25 | 25 | 24 | 25 | 24 | 24 | 27 | 25 | 26 | 24 |
| **Height (cm)** | 174 | 175 | 177 | 189 | 185 | 171 | 173 | 169 | 160 | 164 |
| **Dominant Hand** | Right | Right | Right | Right | Left | Right | Right | Right | Right | Left |

Appendix Table 2. Stabbing experiment 2 volunteer body data.

| **Participant Number** | **11** | **12** | **13** | **14** | **15** | **16** | **17** | **18** | **19** | **20** |
| --- | --- | --- | --- | --- | --- | --- | --- | --- | --- | --- |
| **Healthy Body Fat (%)** | 16.3 | 22.9 | 7.3 | 17.5 | 16.1 | 29.3 | 26.4 | 26.5 | 34 | 26.8 |
| **Weight (kg)** | 77.2 | 85.5 | 74.2 | 90.9 | 72 | 61.1 | 64.7 | 59.8 | 73.4 | 49 |
| **Healthy Body Water (%)** | 60.8 | 54.5 | 65.5 | 58.4 | 59 | 51.6 | 52.8 | 54.5 | 49.1 | 53.4 |
| **Visceral Fat** | 8 | 10 | 2 | 4 | 3 | 5 | 2 | 2 | 4 | 2 |
| **Muscle Mass** | 61.4 | 62.7 | 65.4 | 71.3 | 57.4 | 41.1 | 45.2 | 41.8 | 46 | 34 |
| **Physique Rating** | 5 | 2 | 8 | 5 | 4 | 5 | 5 | 5 | 1 | 5 |
| **Bone Mass (kg)** | 3.2 | 3.3 | 3.4 | 3.7 | 3 | 2.2 | 2.4 | 2.2 | 2.5 | 1.8 |
| **BMR (kcal)** | 1848 | 1917 | 1968 | 2229 | 1770 | 1289 | 1431 | 1358 | 1477 | 1124 |
| **Metabolic Age** | 37 | 46 | 20 | 22 | 21 | 34 | 24 | 19 | 44 | 18 |
| **Energy (kJ)** | 7732 | 8020 | 8234 | 9326 | 7405 | 5393 | 5987 | 5681 | 6179 | 4702 |
| **Age** | 52 | 47 | 35 | 24 | 27 | 49 | 30 | 23 | 32 | 28 |
| **Height (cm)** | 178 | 180 | 188 | 191 | 185 | 167 | 178 | 158 | 183 | 152 |
| **Dominant Hand** | Right | Right | Right | Right | Right | Right | Right | Left | Right | Right |

## Supplementary Tables 3, 4 and 5: The penetrative forces, blunt edge radius, tip radius and blunt tip angle for each instrument for each simulant.

Appendix Table 3. Skin simulant

|  | Penetration Force (N) | Penetration Force (N) | Penetration Force (N) | Average Penetration Force (N) | Blunt edge radius (mm) | Tip radius (mm) | Blunt edge tip angle (˚) |
| --- | --- | --- | --- | --- | --- | --- | --- |
| All-purpose 1 | 13.65 | 10.65 | 10.65 | 11.7 | 0.01 | 0.03 | 109.4 |
| All-purpose 2 | 9.35 | 13.85 | 15.15 | 12.8 | 0.01 | 0.04 | 114.77 |
| All-purpose 3 | 13.15 | 13.5 | 13.15 | 13.3 | 0.01 | 0.06 | 155.31 |
| All-purpose 4 | 11.15 | 13.15 | 12.15 | 12.1 | 0.01 | 0.05 | 112.73 |
| All-purpose 5 | 12.65 | 13.65 | 13.5 | 13.3 | 0.03 | 0.06 | 111.1 |
| Steak 1 | 20.35 | 19 | 22.5 | 20.6 | 0.02 | 0.05 | 124.77 |
| Steak 2 | 22.5 | 18.85 | 20.15 | 20.5 | 0.05 | 0.25 | 149.82 |
| Steak 3 | 20.35 | 20.35 | 18.15 | 19.6 | 0.03 | 0.01 | 111.49 |
| Steak 4 | 18 | 18.5 | 17.35 | 18 | 0.02 | 0.02 | 126.13 |
| Steak 5 | 20.65 | 20.15 | 20.65 | 20.5 | 0.04 | 0.02 | 92.62 |
| Instrumented Knife | 13.85 | 12.65 | 13.65 | 13.4 | 0.01 | 0.01 | 108.15 |
| Flat-head Screwdriver | 63.15 | 78 | 78.64 | 73.3 | n/a | n/a | n/a |
| Phillips Screwdriver | 60 | 62.48 | 67.65 | 63.4 | n/a | n/a | n/a |

Appendix Table 4. Pork leg

|  | Penetration Force (N) | Penetration Force (N) | Penetration Force (N) | Average penetration Force (N) | Blunt edge radius (mm) | Tip radius (mm) | Blunt edge tip angle (˚) |
| --- | --- | --- | --- | --- | --- | --- | --- |
| All-purpose 1 | 13.65 | 13.65 | 11.35 | 12.9 | 0.01 | 0.03 | 109.4 |
| All-purpose 2 | 13 | 7.85 | 9.15 | 10.00 | 0.01 | 0.04 | 114.77 |
| All-purpose 3 | 10.65 | 7.35 | 8.35 | 8.8 | 0.01 | 0.06 | 155.31 |
| All-purpose 4 | 14.35 | 13.35 | 13.5 | 13.7 | 0.01 | 0.05 | 112.73 |
| All-purpose 5 | 17.35 | 13.65 | 14.15 | 15.1 | 0.03 | 0.06 | 111.1 |
| Steak 1 | 44.65 | 43.85 | 40.85 | 41.5 | 0.02 | 0.05 | 124.77 |
| Steak 2 | 41.85 | 42.65 | 39.85 | 33.7 | 0.05 | 0.25 | 149.82 |
| Steak 3 | 33.85 | 33.85 | 33.35 | 33.7 | 0.03 | 0.01 | 111.49 |
| Steak 4 | 41.65 | 49.35 | 65.7 | 52.2 | 0.02 | 0.02 | 126.13 |
| Steak 5 | 49 | 45.35 | 51.98 | 48.8 | 0.04 | 0.02 | 92.62 |
| Instrumented Knife | 25.5 | 25.35 | 25.35 | 25.4 | 0.01 | 0.01 | 108.15 |
| Flat-head Screwdriver | Not achieved | Not achieved | Not achieved | Not achieved | n/a | n/a | n/a |
| Phillips Screwdriver | 219.5 | 247.3 | 236 | 234.3 | n/a | n/a | n/a |

Appendix Table 5. Pork ribs

|  | Penetration Force (N) | Penetration Force (N) | Penetration Force (N) | Average Penetration Force (N) | Blunt edge radius (mm) | Tip radius (mm) | Blunt edge tip angle (˚) |
| --- | --- | --- | --- | --- | --- | --- | --- |
| All-purpose 1 | 17 | 16.9 | 7.65 | 13.8 | 0.01 | 0.03 | 109.4 |
| All-purpose 2 | 10 | 9.85 | 11.35 | 10.4 | 0.01 | 0.04 | 114.77 |
| All-purpose 3 | 10.35 | 10.35 | 10.35 | 10.4 | 0.01 | 0.06 | 155.31 |
| Sabatier 4 | 12 | 9 | 10.15 | 10.4 | 0.01 | 0.05 | 112.73 |
| All-purpose 5 | 16.65 | 13.85 | 14.65 | 15.1 | 0.03 | 0.06 | 111.1 |
| All-purpose 1 | 43.35 | 37.65 | 41.65 | 40.9 | 0.02 | 0.05 | 124.77 |
| Steak 2 | 36.65 | 32 | 51.68 | 40.1 | 0.05 | 0.25 | 149.82 |
| Steak 3 | 45.85 | 54.15 | 46.35 | 48.8 | 0.03 | 0.01 | 111.49 |
| Steak 4 | 47.65 | 40.35 | 39.85 | 42.6 | 0.02 | 0.02 | 126.13 |
| Steak 5 | 34.85 | 39.85 | 37 | 37.2 | 0.04 | 0.02 | 92.62 |
| Instrumented Knife | 28.65 | 32 | 32 | 30.9 | 0.01 | 0.01 | 108.15 |
| Flat-head Screwdriver | Not achieved | Not achieved | Not achieved | Not achieved | n/a | n/a | n/a |
| Phillips Screwdriver | Not achieved | Not achieved | Not achieved | Not achieved | n/a | n/a | n/a |

## Supplementary Table 6: The mean, minimum, maximum and standard deviation of penetrative forces (Newtons) for each instrument for each simulant for stabbing experiment 1. * A minimal value is not presented for the Sabatier knife due to the breaking of the Sabatier knife.

| **Weapon** | **Material** | **Gender** | **Hand** | **Mean** | **Std. Dev.** | **Min** | **Max** |
| --- | --- | --- | --- | --- | --- | --- | --- |
| **Flat-head Screwdriver** | **Skin Simulant** | Male | Non-Dom | 203 | 67 | 102 | 260 |
|  |  |  | Dom | 258 | 79 | 153 | 356 |
|  |  | Female | Non-Dom | 85 | 29 | 48 | 123 |
|  |  |  | Dom | 116 | 40 | 53 | 159 |
|  | **Pork Leg** | Male | Non-Dom | 211 | 91 | 135 | 360 |
|  |  |  | Dom | 193 | 77 | 134 | 314 |
|  |  | Female | Non-Dom | 89 | 18 | 62 | 107 |
|  |  |  | Dom | 98 | 22 | 77 | 133 |
|  | **Pork Rib** | Male | Non-Dom | 156 | 21 | 129 | 187 |
|  |  |  | Dom | 193 | 77 | 134 | 314 |
|  |  | Female | Non-Dom | 95 | 22 | 63 | 120 |
|  |  |  | Dom | 107 | 26 | 71 | 131 |
| **Phillips Screwdriver** | **Skin Simulant** | Male | Non-Dom | 222 | 72 | 136 | 335 |
|  |  |  | Dom | 264 | 77 | 187 | 372 |
|  |  | Female | Non-Dom | 98 | 31 | 53 | 132 |
|  |  |  | Dom | 108 | 35 | 54 | 146 |
|  | **Pork Le** | Male | Non-Dom | 182 | 67 | 116 | 288 |
|  |  |  | Dom | 198 | 105 | 110 | 346 |
|  |  | Female | Non-Dom | 82 | 15 | 61 | 102 |
|  |  |  | Dom | 94 | 30 | 47 | 130 |
|  | **Pork Rib** | Male | Non-Dom | 179 | 82 | 116 | 316 |
|  |  |  | Dom | 190 | 86 | 127 | 335 |
|  |  | Female | Non-Dom | 88 | 23 | 49 | 103 |
|  |  |  | Dom | 102 | 26 | 63 | 136 |
| **All-purpose Knife** | **Skin Simulant** | Male | Non-Dom | 184 | 44 | 134 | 254 |
|  |  |  | Dom | 200 | 33 | 161 | 251 |
|  |  | Female | Non-Dom | 85 | 39 | 42 | 128 |
|  |  |  | Dom | 95 | 40 | 35 | 125 |
|  | **Pork Leg** | Male | Non-Dom | 149 | 43 | 97 | 207 |
|  |  |  | Dom | 159 | 63 | 108 | 256 |
|  |  | Female | Non-Dom | 64 | 43 | * | 104 |
|  |  |  | Dom | 70 | 49 | * | 114 |
|  | **Pork Rib** | Male | Non-Dom | 61 | 85 | * | 175 |
|  |  |  | Dom | 51 | 70 | * | 139 |
|  |  | Female | Non-Dom | 60 | 55 | * | 112 |
|  |  |  | Dom | 73 | 69 | * | 150 |
| **Steak Knife** | **Skin Simulant** | Male | Non-Dom | 170 | 50 | 118 | 243 |
|  |  |  | Dom | 168 | 50 | 133 | 255 |
|  |  | Female | Non-Dom | 77 | 29 | 41 | 117 |
|  |  |  | Dom | 89 | 31 | 41 | 115 |
|  | **Pork Leg** | Male | Non-Dom | 157 | 62 | 99 | 244 |
|  |  |  | Dom | 163 | 66 | 112 | 268 |
|  |  | Female | Non-Dom | 67 | 15 | 51 | 86 |
|  |  |  | Dom | 80 | 37 | 41 | 116 |
|  | **Pork Rib** | Male | Non-Dom | 118 | 20 | 88 | 141 |
|  |  |  | Dom | 126 | 25 | 104 | 158 |
|  |  | Female | Non-Dom | 81 | 27 | 40 | 104 |
|  |  |  | Dom | 90 | 29 | 56 | 128 |

## Supplementary Table 7: The mean, minimum, maximum and standard deviation of penetrative forces (Newtons) for each instrument for pork thigh and pork ribs for stabbing experiment 2.

| **Weapon** | **Strength** | **Material** | **Gender** | **Hand** | **Mean** | **Std. Dev.** | **Min** | **Max** |
| --- | --- | --- | --- | --- | --- | --- | --- | --- |
| **Flat-head**  **Screwdriver** | **Mild** | **Pork Leg** | Male | Non- Dom | 103.96 | 49.79 | 34.72 | 164.11 |
|  |  |  |  | Dom | 114.58 | 51.62 | 46.82 | 161.06 |
|  |  |  | Female | Non- Dom | 50.97 | 36.56 | 12.17 | 106.44 |
|  |  |  |  | Dom | 56.60 | 49.56 | 12.14 | 139.82 |
|  |  | **Pork Rib** | Male | Non- Dom | 80.72 | 36.95 | 38.25 | 136.73 |
|  |  |  |  | Dom | 87.04 | 35.46 | 45.03 | 141.67 |
|  |  |  | Female | Non- Dom | 49.46 | 43.31 | 14.80 | 120.90 |
|  |  |  |  | Dom | 58.56 | 44.62 | 13.41 | 126.29 |
|  | **Moderate** | **Pork Leg** | Male | Non- Dom | 155.46 | 39.56 | 86.38 | 182.55 |
|  |  |  |  | Dom | 166.32 | 27.96 | 126.27 | 190.93 |
|  |  |  | Female | Non- Dom | 87.34 | 64.90 | 16.94 | 174.88 |
|  |  |  |  | Dom | 98.93 | 87.96 | 24.77 | 198.47 |
|  |  | **Pork Rib** | Male | Non- Dom | 152.14 | 29.48 | 115.84 | 179.72 |
|  |  |  |  | Dom | 217.89 | 46.32 | 186.85 | 296.18 |
|  |  |  | Female | Non- Dom | 81.57 | 63.35 | 28.67 | 184.99 |
|  |  |  |  | Dom | 89.70 | 51.01 | 33.58 | 164.76 |
|  | **Severe** | **Pork Leg** | Male | Non- Dom | 203.36 | 42.81 | 171.42 | 278.51 |
|  |  |  |  | Dom | 243.35 | 22.38 | 211.41 | 267.16 |
|  |  |  | Female | Non- Dom | 131.55 | 56.35 | 54.33 | 199.63 |
|  |  |  |  | Dom | 152.17 | 55.89 | 97.18 | 226.23 |
|  |  | **Pork Rib** | Male | Non- Dom | 210.38 | 40.07 | 169.72 | 261.06 |
|  |  |  |  | Dom | 217.89 | 46.32 | 186.85 | 296.18 |
|  |  |  | Female | Non- Dom | 119.37 | 57.52 | 48.76 | 208.97 |
|  |  |  |  | Dom | 131.47 | 75.01 | 54.69 | 253.87 |

| **Phillips Screwdriver** | **Mild** | **Pork Leg** | Male | Non- Dom | 105.97 | 64.17 | 18.73 | 178.63 |
| --- | --- | --- | --- | --- | --- | --- | --- | --- |
|  |  |  |  | Dom | 151.85 | 43.47 | 77.41 | 185.11 |
|  |  |  | Female | Non- Dom | 56.83 | 38.65 | 10.78 | 102.56 |
|  |  |  |  | Dom | 66.47 | 49.51 | 14.76 | 128.90 |
|  |  | **Pork Rib** | Male | Non- Dom | 82.15 | 32.67 | 42.42 | 128.64 |
|  |  |  |  | Dom | 77.19 | 37.60 | 23.66 | 121.52 |
|  |  |  | Female | Non- Dom | 48.85 | 40.97 | 13.25 | 118.42 |
|  |  |  |  | Dom | 51.68 | 38.77 | 14.05 | 114.90 |
|  | **Moderate** | **Pork Leg** | Male | Non- Dom | 145.53 | 39.36 | 79.51 | 176.89 |
|  |  |  |  | Dom | 151.85 | 43.47 | 77.41 | 185.11 |
|  |  |  | Female | Non- Dom | 93.91 | 72.74 | 18.91 | 180.57 |
|  |  |  |  | Dom | 93.76 | 69.77 | 25.98 | 168.63 |
|  |  | **Pork Rib** | Male | Non- Dom | 144.75 | 34.85 | 101.38 | 173.82 |
|  |  |  |  | Dom | 139.45 | 23.37 | 110.78 | 163.50 |
|  |  |  | Female | Non- Dom | 83.20 | 55.63 | 34.17 | 175.10 |
|  |  |  |  | Dom | 94.63 | 71.98 | 28.45 | 214.46 |
|  | **Severe** | **Pork Leg** | Male | Non- Dom | 197.82 | 46.74 | 134.81 | 263.20 |
|  |  |  |  | Dom | 226.37 | 60.91 | 168.80 | 297.26 |
|  |  |  | Female | Non- Dom | 136.49 | 71.79 | 39.58 | 201.25 |
|  |  |  |  | Dom | 152.64 | 78.85 | 68.82 | 260.42 |
|  |  | **Pork Rib** | Male | Non- Dom | 206.08 | 51.73 | 148.74 | 273.20 |
|  |  |  |  | Dom | 261.71 | 118.83 | 163.31 | 460.53 |
|  |  |  | Female | Non- Dom | 109.27 | 46.74 | 52.98 | 180.33 |
|  |  |  |  | Dom | 155.03 | 96.91 | 63.33 | 319.40 |

| **Weapon** | **Strength** | **Material** | **Gender** | **Hand** | **Mean** | **Std. Dev.** | **Min** | **Max** |
| --- | --- | --- | --- | --- | --- | --- | --- | --- |
| **All-purpose Knife** | **Mild** | **Pork Leg** | Male | Non-Dom | 43.36 | 29.44 | 9.98 | 74.94 |
|  |  |  |  | Dom | 47.40 | 32.25 | 7.44 | 83.59 |
|  |  |  | Female | Non-Dom | 33.74 | 39.29 | 5.22 | 101.79 |
|  |  |  |  | Dom | 30.12 | 30.48 | 7.03 | 81.48 |
|  |  | **Pork Rib** | Male | Non-Dom | 46.48 | 22.29 | 19.75 | 81.22 |
|  |  |  |  | Dom | 57.04 | 37.87 | 13.32 | 116.56 |
|  |  |  | Female | Non-Dom | 42.65 | 53.39 | 4.56 | 135.67 |
|  |  |  |  | Dom | 41.97 | 55.98 | 5.73 | 140.73 |
|  | **Moderate** | **Pork Leg** | Male | Non-Dom | 124.26 | 38.84 | 81.29 | 174.30 |
|  |  |  |  | Dom | 124.83 | 44.20 | 60.96 | 174.10 |
|  |  |  | Female | Non-Dom | 60.11 | 52.52 | 6.11 | 123.57 |
|  |  |  |  | Dom | 65.08 | 65.17 | 8.39 | 163.74 |
|  |  | **Pork Rib** | Male | Non-Dom | 128.10 | 30.23 | 101.15 | 174.04 |
|  |  |  |  | Dom | 160.64 | 66.77 | 90.74 | 259.30 |
|  |  |  | Female | Non-Dom | 67.62 | 60.76 | 6.74 | 157.14 |
|  |  |  |  | Dom | 74.66 | 78.67 | 6.91 | 204.32 |
|  | **Severe** | **Pork Leg** | Male | Non-Dom | 207.42 | 82.69 | 113.08 | 338.02 |
|  |  |  |  | Dom | 206.18 | 75.66 | 134.15 | 327.11 |
|  |  |  | Female | Non-Dom | 100.19 | 62.12 | 17.03 | 184.44 |
|  |  |  |  | Dom | 130.80 | 77.95 | 43.62 | 217.76 |
|  |  | **Pork Rib** | Male | Non-Dom | 203.53 | 44.05 | 138.77 | 248.63 |
|  |  |  |  | Dom | 223.75 | 66.19 | 133.85 | 319.90 |
|  |  |  | Female | Non-Dom | 118.56 | 76.20 | 28.34 | 237.26 |
|  |  |  |  | Dom | 124.69 | 87.14 | 32.79 | 268.18 |

| **Weapon** | **Strength** | **Material** | **Gender** | **Hand** | **Mean** | **Std. Dev.** | **Min** | **Max** |
| --- | --- | --- | --- | --- | --- | --- | --- | --- |
| **Steak Knife** | **Mild** | **Pork Leg** | Male | Non-Dom | 55.70 | 31.87 | 12.40 | 93.66 |
|  |  |  |  | Dom | 61.24 | 35.71 | 22.73 | 103.76 |
|  |  |  | Female | Non-Dom | 35.18 | 19.85 | 16.66 | 66.34 |
|  |  |  |  | Dom | 30.13 | 17.61 | 14.07 | 58.14 |
|  |  | **Pork Rib** | Male | Non-Dom | 38.32 | 14.82 | 17.31 | 56.49 |
|  |  |  |  | Dom | 42.27 | 22.08 | 12.48 | 71.50 |
|  |  |  | Female | Non-Dom | 40.79 | 40.15 | 9.18 | 111.00 |
|  |  |  |  | Dom | 36.84 | 32.43 | 7.88 | 91.63 |
|  | **Moderate** | **Pork Leg** | Male | Non-Dom | 94.74 | 28.96 | 43.91 | 114.09 |
|  |  |  |  | Dom | 99.32 | 34.38 | 44.77 | 133.72 |
|  |  |  | Female | Non-Dom | 56.94 | 35.72 | 24.30 | 111.74 |
|  |  |  |  | Dom | 55.90 | 38.06 | 24.98 | 115.22 |
|  |  | **Pork Rib** | Male | Non-Dom | 101.58 | 19.07 | 77.11 | 123.89 |
|  |  |  |  | Dom | 103.17 | 24.46 | 63.63 | 123.78 |
|  |  |  | Female | Non-Dom | 61.34 | 37.58 | 22.72 | 117.25 |
|  |  |  |  | Dom | 71.05 | 54.60 | 20.03 | 150.84 |
|  | **Severe** | **Pork Leg** | Male | Non-Dom | 158.91 | 34.03 | 125.48 | 200.78 |
|  |  |  |  | Dom | 164.98 | 38.12 | 132.79 | 210.17 |
|  |  |  | Female | Non-Dom | 82.22 | 39.98 | 37.36 | 127.69 |
|  |  |  |  | Dom | 105.20 | 63.88 | 37.75 | 194.72 |
|  |  | **Pork Rib** | Male | Non-Dom | 130.70 | 29.33 | 100.42 | 174.12 |
|  |  |  |  | Dom | 137.68 | 22.03 | 111.20 | 170.31 |
|  |  |  | Female | Non-Dom | 91.31 | 47.06 | 24.84 | 154.43 |
|  |  |  |  | Dom | 99.47 | 54.50 | 29.05 | 169.20 |

## Supplementary text: The results of the univariate and multivariate statistical analysis.

### Explanation of Statistical analysis used

In order to analyse the data collected mixed effects linear regression was used. The design of the experiments involved collecting multiple independent and continuous variables from each participant. As the main variable here, force, is continuous a mixed effects linear regression model was used. A mixed-effects model comprises both fixed and random effects. With multiple mixed variables being collected from the same participant they cannot be considered independent from each other. Thus each variable is considered inter-dependent rather than independent, and so a simple linear regression model cannot be used. Fixed-effects are those that would traditionally utilise linear regression, and the random effects are associated with individual experimental measures that are drawn at random from the dataset. Mixed effect models describe the relationship between independent variables and a response variable, with coefficients that can vary with respect to one or more grouping variables. In order to resolve this, random effects are added for the participant, random effects having prior distribution unlike the fixed effects which do not. This allows for the assumption of a different reference point for force values for each participant, thus resolving the issue of non-independent.

In science and statistics the p-value is the estimated probability of achieving a test value as close as possible to the value or measurement observed. Therefore a p-value is the likelihood of a result occurring by chance. When the p-value is less than 0.05 it is said to disprove the null hypothesis and the result is considered statistically significant. In terms of statistics, when a scientific study is undertaken the observed data is expected to agree with the null hypothesis. The null hypothesis is a default statement that there is no relationship between two measured occurrences, i.e. the results could just have occurred by chance. If the null hypothesis is rejected then this suggests that there is a real relationship between the measured effects, i.e. they are influenced by each other. So if the p-value is less than 0.05 then the variables are considered to have a statistically significant difference. If p ≥0.05 then there is considered to be no significant difference between the two variables being investigated. The tables below show the variable that was tested for e.g. the category sex would consider the results between male and female, the coefficient (gradient) of the line in the linear analysis and the p value gives the significance. The 95% confidence limit shows the possible range for the value of coefficient.

### Forces generated by males and females

#### Stabbing experiment 1

| **Force** | **Coef.** | **P** | **[95% Conf. Interval]** | |
| --- | --- | --- | --- | --- |
| **Sex** | -87.20 | 0.00 | -126.75 | -47.65 |

Univariate analysis of gender

| **Force** | **Coef.** | **P** | **[95% Conf. Interval]** | |
| --- | --- | --- | --- | --- |
| **Weapon 1** | -2.47 | 0.77 | -18.76 | 13.81 |
| **Weapon 2** | -48.78 | 0.00 | -65.06 | -32.49 |
| **Weapon 3** | -37.45 | 0.00 | -53.74 | -21.17 |
| **Sex** | -87.20 | 0.00 | -126.75 | -47.65 |
| **Hand** | 14.83 | 0.01 | 3.31 | 26.34 |
| **Material 1** | -20.95 | 0.00 | -35.05 | -6.85 |
| **Material 2** | -40.77 | 0.00 | -54.87 | -26.66 |

Multivariate analysis of Sex, Weapon 1 (flat-head screwdriver), Weapon 2 (All-purpose knife), Weapon 3 (steak knife), Hand, Material 1 (pork leg) and Material 2 (pork rib)

#### Stabbing experiment 2

| **Force** | **Coef.** | **P** | **[95% Conf. Interval]** | |
| --- | --- | --- | --- | --- |
| **Sex** | -76.12 | 0.00 | -115.62 | -36.63 |

Univariate analysis of Sex

| **Force** | **Coef.** | **P** | **[95% Conf. Interval]** | |
| --- | --- | --- | --- | --- |
| **Weapon 1** | -1.40 | 0.84 | -14.89 | 12.09 |
| **Weapon 2** | -36.31 | 0.00 | -49.81 | -22.82 |
| **Weapon 3** | -45.63 | 0.00 | -59.12 | -32.14 |
| **Sex** | -76.12 | 0.00 | -115.62 | -36.63 |
| **Hand** | 15.84 | 0.00 | 6.30 | 25.38 |
| **Material** | -11.75 | 0.02 | -21.28 | -2.21 |

Multivariate analysis of Sex, Weapon 1 (flat-head screwdriver), Weapon 2 (All-purpose knife), Weapon 3 (steak knife), Hand and Material

### Forces generated by dominant and non-dominant hands

#### Combined data

| **Force** | **Coef.** | **P** | **[95% Conf. Interval]** | |
| --- | --- | --- | --- | --- |
| **Hand** | 15.84 | 0.00 | 5.16 | 26.52 |

Univariate analysis of Hand

| **Force** | **Coef.** | **P** | **[95% Conf. Interval]** | |
| --- | --- | --- | --- | --- |
| **Weapon 1** | -1.40 | 0.84 | -14.89 | 12.09 |
| **Weapon 2** | -36.31 | 0.00 | -49.81 | -22.82 |
| **Weapon 3** | -45.63 | 0.00 | -59.12 | -32.14 |
| **Sex** | -76.12 | 0.00 | -115.62 | -36.63 |
| **Hand** | 15.84 | 0.00 | 6.30 | 25.38 |
| **Material** | -11.75 | 0.02 | -21.28 | -2.21 |

Multivariate analysis of Hand, Weapon 1 (flat-head screwdriver), Weapon 2 (All-purpose knife), Weapon 3 (steak knife), Sex and Material

### Forces generated by knives and screwdrivers

| **Force** | **Coef.** | **P** | **[95% Conf. Interval]** | |
| --- | --- | --- | --- | --- |
| **Screwdriver** | -1.40 | 0.80 | -12.36 | 9.56 |

Univariate analysis of Screwdrivers

| **Force** | **Coef.** | **P** | **[95% Conf. Interval]** | |
| --- | --- | --- | --- | --- |
| **Screwdriver** | -1.40 | 0.79 | -11.69 | 8.89 |
| **Sex** | -91.00 | 0.00 | -137.50 | -44.50 |
| **Hand** | 22.02 | 0.00 | 11.73 | 32.30 |
| **Material** | -6.30 | 0.23 | -16.58 | 3.99 |

Multivariate analysis of Screwdrivers, Sex, Hand and Material
